# Topography of Functional Organization of Beat Perception in Human Premotor Cortex: Causal Evidence From a Transcranial Magnetic Stimulation (TMS) Study

**DOI:** 10.1002/hbm.70225

**Published:** 2025-05-09

**Authors:** Giorgio Lazzari, Giulio Costantini, Stefania La Rocca, Andrea Massironi, Luigi Cattaneo, Virginia Penhune, Carlotta Lega

**Affiliations:** ^1^ Department of Brain and Behavioral Sciences University of Pavia Pavia Italy; ^2^ Department of Psychology University of Milano‐Bicocca Milan Italy; ^3^ Center for Mind/Brain Sciences (CIMeC) University of Trento Trento Italy; ^4^ Psychology Department Concordia University Montreal Canada; ^5^ Montreal Laboratory for Brain, Music and Sound (BRAMS) The Centre for Research in Brain, Language and Music (CRBLM) Montreal Canada

**Keywords:** music, premotor cortex, rhythm perception, supplementary motor areas, transcranial magnetic stimulation

## Abstract

Humans can flexibly extract a regular beat from complex rhythmic auditory patterns, as often occurs in music. Contemporary models of beat perception suggest that the premotor cortex (PMC) and the supplementary motor area (SMA) are integral to this process. However, how these motor planning regions actively contribute to beat perception, along with any potential hemispheric specialization, remains open questions. Therefore, following the validation of stimuli in a behavioral experiment (Experiment I, *N* = 29, 12 males, mean age = 23.8 ± 0.7 years), we employed transcranial magnetic stimulation (TMS) to test the causal contribution of these regions to beat perception. In Experiment II (*N* = 40, 16 males, mean age = 23.2 ± 2.37 years), we applied online repetitive TMS (rTMS) over a defined grid encompassing the right rostral and caudal dPMC, SMA, and pre‐SMA, and a sham control location. Results showed that stimulation of the caudal portion of right dPMC selectively affected beat perception compared to all other regions. In Experiment III (preregistered, *N* = 42, 17 males, mean age = 23.5 ± 2.61 years), we tested the lateralization of this contribution by applying rTMS over right and left caudal dPMC. Our results showed that only stimulation over right, but not left, dPMC modulated beat perception. Finally, across all three experiments, individual differences in musical reward predicted beat perception sensitivity. Together, these results support the causal role of the right dPMC in generating internal action predictions and perceptual expectations regarding ongoing sequential events, in line with recent models emphasizing the role of the dorsal auditory stream in beat‐based temporal perception. These findings offer valuable insights into the functional organization of the premotor cortex, contributing to a deeper understanding of the neural mechanisms involved in human rhythm perception.

## Graphical abstract

This study shows that the right dorsal premotor cortex (dPMC) plays a causal role in human rhythm perception, even without overt movement. Using transcranial magnetic stimulation (TMS), we found that right dPMC stimulation affected beat perception, highlighting its unique role in building internal timing models for rhythm perception.

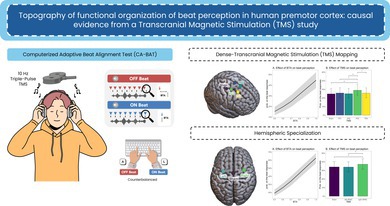

## 1 Introduction

Beat perception is the capacity to detect a regular pulse, or beat, in rhythmic auditory patterns (Honing 2013; Large and Palmer 2002; London 2012). The ability to perceive the beat of musical rhythms emerges spontaneously in infancy, does not require special training, and is present in every culture (Winkler et al. 2009; Savage et al. 2015). Even when people listen passively to rhythmic stimuli, neuroimaging studies consistently demonstrate the engagement of the dorsal auditory‐motor network (Proksch et al. 2020; Chen et al. 2008a; Grahn and Brett 2007; Bengtsson et al. 2009; Grahn 2009; Grahn and Rowe 2013). This network links auditory regions in the posterior temporal lobe with parietal, premotor (PMC), supplementary motor area (SMA), and motor (M1) cortical areas, as well as subcortical regions like the cerebellum and basal ganglia. The dorsal auditory stream is crucial for integrating auditory and motor information both in language and music (Hickok and Poeppel 2004; Rauschecker 2011; Chen et al. 2008b; Lega et al. 2016).

Based on this evidence, several authors have argued that motor planning regions such as PMC and SMA may be critical in generating temporal predictions and transferring this information to auditory regions to guide perception (Arnal 2012; Patel and Iversen 2014; Ross et al. 2016; Morillon and Baillet 2017; Araneda et al. 2017). The Patel and Iversen's (2014) “Action Simulation for Auditory Prediction” (ASAP) hypothesis suggests that motor planning regions simulates actions to generate temporal predictions regarding beat times. This information is then transferred from motor to auditory regions where it provides temporal predictive signals for upcoming beats, therefore shaping the perceptual interpretation of musical rhythms. In other words, rather than being a passive consequence of the association between beat perception and movement, motor planning activity during beat perception might play an active role in the formation and maintenance of internal predictive models that guide beat perception (Cannon and Patel 2021; Ross et al. 2016, 2018a, 2018b; Patel and Iversen 2014). At the neural level, the ASAP hypothesis posits that communication between motor and auditory areas during beat perception relies on a dorsal auditory pathway, specifically involving the dorsal PMC. A recent extension of this model proposes that the motor system contributes to the accuracy of auditory predictions by providing a periodic temporal framework through oscillatory connections between SMA and the dorsal striatum, making this circuit crucial to beat maintenance and to auditory expectations (Cannon and Patel 2021).

Neuroimaging studies cited above provide evidence supporting the role of PMC and SMA in beat perception (Proksch et al. 2020; Chen et al. 2008a; Grahn and Brett 2007; Bengtsson et al. 2009; Grahn 2009; Grahn and Rowe 2013). However, the causal role of these regions remains unclear. Indeed, research on the effects of brain stimulation on rhythm perception has produced mixed findings, with outcomes varying based on the stimulation technique, target region, and task design. Leow et al. (2022) used transcranial direct current stimulation (tDCS) to modulate excitability in the supplementary motor area (SMA) and found that increasing SMA excitability enhanced rhythm discrimination, while decreasing it impaired performance. This study asked participants to make same/different judgments of short rhythmic sequences, thus employing a 2‐alternative forced choice (2‐AFC) response method, which likely engaged additional cognitive processes, such as working memory and comparison, beyond rhythm perception itself. Using an adaptive version of the beat alignment task with real music excerpts, Ross et al. (2018a) applied transcranial magnetic stimulation (TMS) using a continuous theta burst stimulation (cTBS) protocol and found that stimulation over the left posterior parietal cortex (PPC) selectively impaired the ability to detect shifts in beat phase but had no effect on tempo perception. Notably, cTBS over the left SMA did not produce any significant effects. A subsequent study using the same tasks (2018b) demonstrated that cTBS over the left dPMC interfered with tempo discrimination but did not impact phase perception. Each of these studies focused on isolated targets within the premotor cortex (SMA or dPMC) and did not compare between regions. Furthermore, the use of different stimulation techniques (e.g., tDCS vs. TMS) and protocols (e.g., offline theta burst stimulation)—make cross‐study comparisons challenging. To address these gaps, our study aims to directly compare the effects of stimulation over SMA and dPMC with the same paradigm. Moreover, we avoid the limitations of a 2‐AFC design by employing a task that assesses rhythm perception without requiring memory‐based comparisons, allowing us to isolate perceptual processes more effectively.

Evidence about possible lateralization in rhythm perception and perceptual timing tasks is also mixed (see Kasdan et al. 2022 for a review). While several studies emphasize the right hemisphere's role in the dorsal auditory stream for both beat perception (Chen et al. 2008a; Siman‐Tov et al. 2022) and beat production (Giovannelli et al. 2014; Kung et al. 2013), others have demonstrated more bilateral or left‐lateralized involvement (Ross et al. 2018a, 2018b; Pollok et al. 2008, 2017). Given these discrepancies, we opted to first target the right hemisphere, as the dominant role of the right dorsal stream has been widely suggested in auditory‐motor integration, particularly in the context of music (Jünemann et al. 2023; Vaquero et al. 2018). Additionally, previous studies have shown that nonmusicians display stronger right‐hemisphere activation during rhythmic tasks compared to expert musicians (Chen et al. 2008a; Limb et al. 2006; Grahn and McAuley 2009), making it relevant to examine this effect in a general population.

Based on this evidence, the objective of the current studies was to use TMS to systematically examine the causal contribution of the PMC and SMA to beat perception and to test the relative contributions of the right and left hemispheres. To do this, we first conducted a behavioral study (Experiment I) to validate the beat perception paradigm (adapted from Harrison and Müllensiefen 2018a) and used this data to create a model of predicted performance for the TMS studies. Then, we applied online repetitive TMS (rTMS) to a grid of four targets covering right caudal and rostral dPMC and medial pre‐SMA and SMA while participants completed the beat perception task (Experiment II). We utilized a dense TMS mapping approach developed in our earlier work which allowed for within‐subject comparisons of each region (Lega, Chelazzi, et al. 2020; Lega, Pirruccio, et al. 2020; Cattaneo 2018; Parmigiani and Cattaneo 2018). The results of Experiment II showed that only stimulation over right caudal dPMC modified beat perception. Therefore, in the preregistered Experiment III we compared inhibitory TMS over left and right caudal dPMC as a confirmatory replication of the original findings and as a test for hemispheric specialization. These results showed that stimulation over right, but not left caudal dPMC modified beat perception. Together, this set of experiments provides evidence for the causal role of the right dPMC in beat perception and distinguishes it from possible contributions of the SMA to other aspects of timing.

Furthermore, recent literature investigates the relationship between reward and rhythm in both production and perception (see Zatorre 2024; Fiveash et al. 2022, 2023), often showing a co‐activation of the motor and reward networks at the neural level (Matthews et al. 2020; Zalta et al. 2024). Additionally, pleasure has been shown to promote more precise rhythmic abilities at a behavioral level (e.g., Smykovskyi et al. 2022; Lazzari et al. 2024). This connection is further supported by studies on musical groove—the pleasurable urge to move to music—which suggest a strong interplay between rhythm complexity, movement, prediction, and pleasure (Janata et al. 2012; Matthews et al. 2020; Vuust and Witek 2014; Witek et al. 2014; Spiech et al. 2022, 2024; Romkey et al. 2025). Building on this evidence, we included a measure of musical reward sensitivity—the Barcelona Music Reward Questionnaire (BMRQ; Mas‐Herrero et al. 2013)—to examine whether individual differences in sensitivity to musical reward influence beat perception and modulate the effects of TMS.

## 2 Experiment I: Behavioral Study

The aim of this behavioral experiment was to validate a modified version of an existing beat perception task to be used in the TMS experiments. Further, we used behavioral data to create a model of performance that could be applied to the TMS data. We included only nonmusicians to avoid a potential confound related to musical expertise. Prior research has shown that musicians and nonmusicians differ in their performance on rhythmic tasks (Spiech et al. 2023; Repp and Doggett 2007; Chen et al. 2008b; Skaansar et al. 2019; Grahn and Rowe 2009; Mosing et al. 2014; Grahn and McAuley 2009; Grahn and Schuit 2012) and engage distinct neural networks during beat perception and production (Pando‐Naude et al. 2021; Criscuolo et al. 2022; Lumaca et al. 2024). To control for expertise‐related variability and maintain a homogeneous sample, we included only participants with less than three years of musical training. The same selection criteria applied to Experiments II and III.

## 3 Materials and Methods

### 3.1 Participants

Twenty‐nine volunteers took part in the study (12 males, mean age = 23.8 ± 0.7 years). All participants were nonmusicians, having received less than 3 years of formal or informal musical training (*M* = 1.10, SD = 1.34). The experimental protocol was approved by the local ethical committee of the University of Milano‐Bicocca (Ethical Committee Prot. No. 560) and participants were treated in accordance with the Declaration of Helsinki.

### 3.2 Stimuli

The stimuli were sourced from the online repository provided by Harrison and Müllensiefen (2018a, 2018b, https://doi.org/10.5281/zenodo.1210808). This bank includes 25 purely instrumental tracks from different musical genres and meters, each lasting approximately 5 s. Each track is overlaid with a metronome beep‐track composed of a 20‐ms sine tone with a frequency of 1000 Hz and a 10‐ms fade‐out. The beeps start at the onset of each musical track, ensuring that every beat has an associated beep. The amount of displacement between the track's beat period and the metronome “beep” is quantified through the beep‐track accuracy (BTA) index. This index has been defined by (Harrison and Müllensiefen 2018a, Equation (2)) as a transformation of *p*, the proportion of displacement of the “beep” with respect to the beat period, using the formula BTA = cos^4^ (πP). The BTA formula has been derived heuristically by Harrison & Müllensiefen to ensure approximate linearity between BTA and the difficulty of the 2‐AFC task. BTA values can be interpreted with reference to corresponding values of *p*. For example, BTA = 1 corresponds to an on‐beat stimulus (*p* = 0, i.e., no displacement), BTA = 0 corresponds to a 50% displacement (*p* = 0.50, i.e., an anti‐phase stimulus). Due to the validation nature of Experiment I, we used seven off‐beat BTAs, 0.50, 0.55, 0.60, 0.65, 0.70, 0.75, 0.80 (corresponding to displacements of 0.18, 0.17, 0.16, 0.15, 0.13, 0.12, and 0.11, respectively) and the on‐beat track (BTA = 1.0) for each of the 25 musical stimuli. To have equal numbers of on‐beat and off‐beat stimuli, each on‐beat version was repeated seven times. Thus, half of the stimuli were on‐beat (1.0 BTA), and the other half were off‐beat, for a total of 350 stimuli. To enhance transparency and clarity, we have included in the Supporting Information one audio example for an on‐beat stimulus (i.e., “baobab”, BTA = 1.0) and one version of the same track for each degree of off‐beat displacement (BTA from 0.5 to 0.8 in 0.5 steps).

### 3.3 Procedure

**FIGURE 1:**
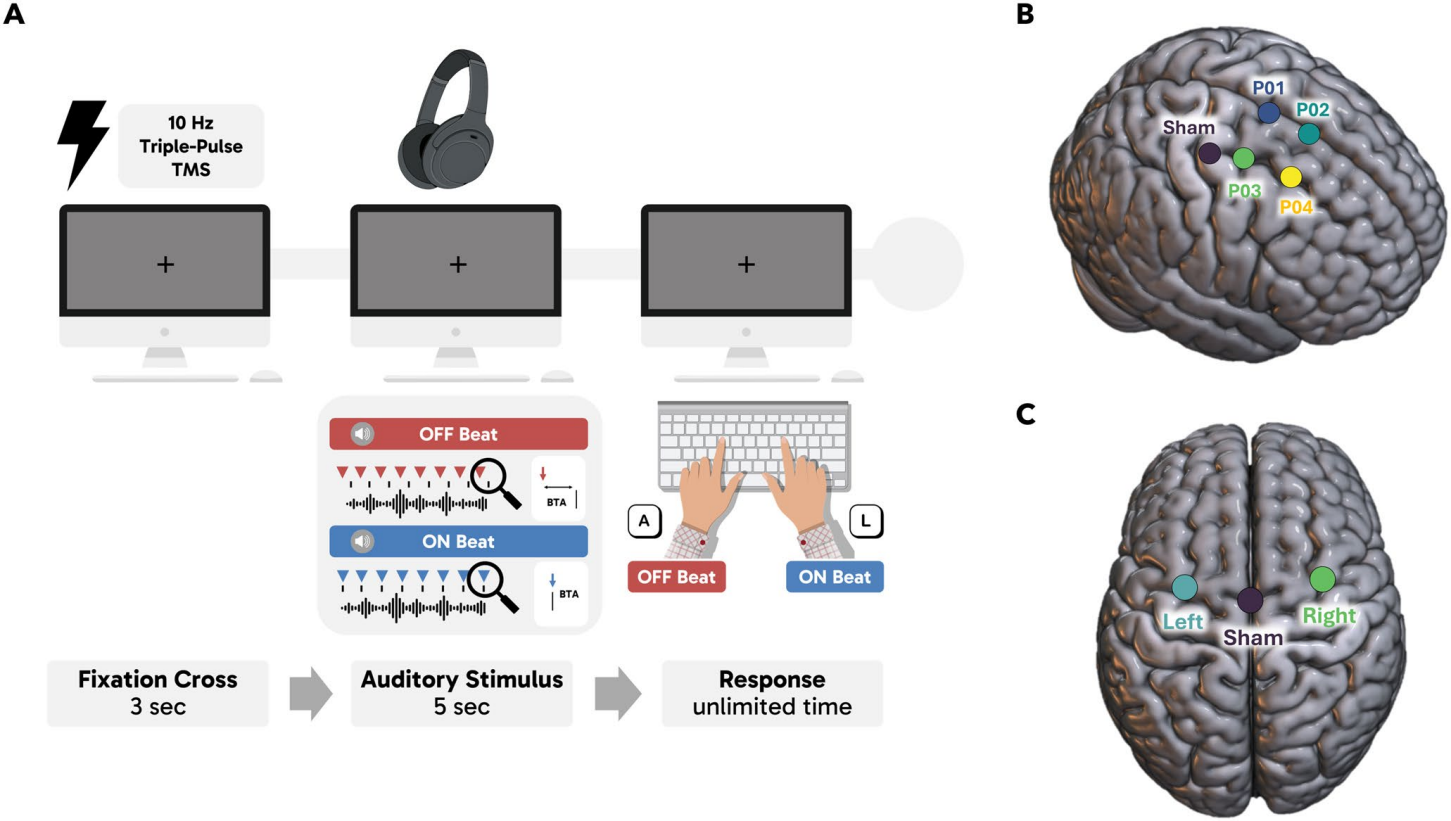
(A) Schematic representation of the task procedure for the behavioral (Exp. I) and TMS experiments (Exp. II and III). After the fixation cross (3 s), participants listened to the musical track (5 s). Participants were asked to categorize each track as on‐beat or off‐beat by pressing the “A” or “L” keys on the keyboard (counterbalanced across participants). A triple‐pulse 10‐Hz TMS was delivered immediately before the auditory stimulus, with the third pulse aligned with the musical track onset, only in Experiment II and III. (B) Surface render of the MNI‐152 template with indication of the average cortical location of each of the four active spots for Experiment II (P01: Right SMA, P02: Right pre‐SMA, P03: Right caudal dPMC, P04: Right rostral dPMC) and the sham control condition. (C) Surface render of the MNI‐152 template with indication of the average cortical location of Experiment III (right and left dPMC) and the sham control condition.

Each trial started with a fixation cross lasting 3 s, after which the auditory stimulus was presented. Participants were asked to categorize each extract as “on‐beat” or “off‐beat” as quickly and accurately as possible by pressing the “A” or “L” keys on the keyboard, as illustrated in Figure 1A. The response keys were counterbalanced across participants. Six practice trials were included at the start, using the same practice tracks from Harrison and Müllensiefen's (2018a, 2018b) original CA‐BAT task. Participants completed three on‐beat and three off‐beat practice trials. To ensure the task was easily understandable, the off‐beat practice trials featured the highest BTA (i.e., BTA = 0.5), representing the easiest off‐beat condition, and participants received the feedback (“incorrect”) after wrong responses. Short breaks were incorporated every 70 trials. The order of presentation of stimuli was randomized. Stimuli were binaurally delivered using professional headphones (Sennheiser HD 280 Pro). The software OpenSesame (Mathôt et al. 2012) was used for stimuli presentation and data collection. In addition to the beat perception test, we also collected information about participants' sensitivity to musical reward using the BMRQ (Mas‐Herrero et al. 2013). The questionnaire comprises 20 questions categorized into five sub‐factors: music‐seeking, emotion evocation, mood regulation, sensorimotor and social reward. Participants are asked to rate their level of agreement with each statement on a 5‐point Likert scale, from (1) “Fully disagree” to (5) “Fully agree.” An overall score (min = 20, max = 100) can be derived by summing up the individual sub‐factor scores. The entire experiment lasted approximately 1 h, including instructions, short breaks, and questionnaires.

### 3.4 Statistical Analysis

The focus of statistical analyses was predicting the binary responses (0 = off‐beat, 1 = on‐beat), using different sets of independent variables. To account for the binary dependent variable and the nested structure of the data (within participant and within trial), we fitted multilevel logistic regression models (Gelman and Hill 2006). All models included random intercepts per participant and per musical track, to account for both individual differences in response styles and stimulus characteristics. Models were fitted using package *lme4* (Bates et al. 2015) in the R statistical language (R Core Team 2023). *R* ^2^ for multilevel models was estimated using package *performance* (Lüdecke 2021; Nakagawa and Schielzeth 2013).

We first examined potential shifts in participants' responses by predicting the binary response from the trial number (rescaled between 0 and 1 to ensure convergence). We then used the likelihood‐ratio test and the Akaike Information Criterion (AIC) to compare two alternative models in which the binary response was predicted by BTA. In the first one (Model 1), BTA was considered a discrete variable with eight levels: BTA = 0.50 (i.e., metronome is maximally off‐beat), BTA = 0.55, BTA = 0.60, BTA = 0.65, BTA = 0.70, BTA = 0.75, BTA = 0.80, and BTA = 1 (i.e., metronome is on‐beat). In the second and simpler model (Model 2), BTA was considered a continuous predictor, ranging between 0.50 and 1. This second model is in line with the original definition of BTA as an index that is linearly related to difficulty (Harrison and Müllensiefen 2018a). Conversely, Model 1 is more flexible and can accommodate non‐linear relationships. By comparing the fit of these two models, we could inspect whether changing the original task (e.g., from a 2‐AFC to a single‐trial paradigm) altered the linearity of the BTA‐difficulty relationship.

An additional interaction analysis (Model 3) was carried out to explore whether individual differences in musical reward—assessed with the BMRQ–were related to the sensitivity of participant responses to BTA. To facilitate interpretation of main effects, BTA was centered on its mean and BMRQ was standardized with *M* = 0 and SD = 1 before entering the model.

To provide an idea of the magnitude of the effects, we also report regression coefficients on the odds‐ratio scale (*e* ^*b*^). These coefficients indicate the change in relative odds of responding “on‐beat” to responding “off‐beat” when the corresponding predictor increases by one unit. Specifically, the odds are multiplied by *e* ^*b*^ for each unit increase in the predictor.

## 4 Results

The complete statistics for all models are reported in the Table S3. The first analysis revealed that participants' response patterns did not change significantly over the 350 trials (*b* = −0.057, *z* = −0.781, *p* = 0.435). Since the model predicts on‐beat responses, this result suggests that participants' likelihood of responding on‐beat remained stable throughout the experiment. This stability is likely due to the balanced number of on‐beat and off‐beat trials. The likelihood‐ratio test revealed no significant differences in how well Model 1 and Model 2 fitted the data (ꭓ^2^ (6) = 2.10, *p* = 0.910), indicating that considering BTA as a discrete predictor (Model 1) did not improve predictions compared to the simpler continuous model (Model 2). The same conclusion was suggested by the AIC (AIC_Model1_ = 12,156, AIC_Model2_ = 12,146). Hence, the more parsimonious Model 2 was preferred.

**FIGURE 2:**
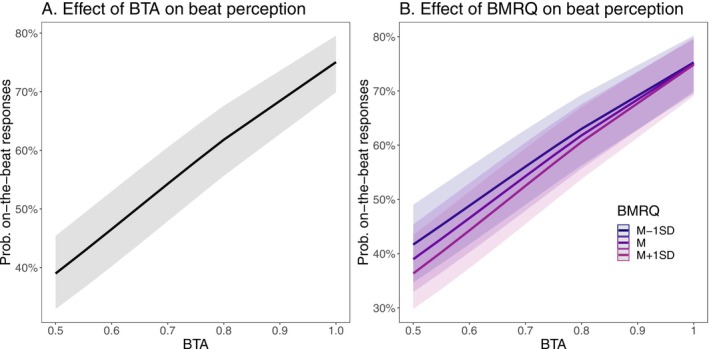
Visualization of the marginal predicted values in terms of on‐beat response probabilities as a function of (A) beep‐track accuracy (BTA) levels and (B) BTA levels and music reward (BMRQ) scores.

Model 2 revealed a significant effect of BTA (*b* = 3.10, *z* = 26.26, *p* < 0.001, *e* ^*b*^ = 22.16; Marginal *R* ^2^ = 0.084, Conditional *R* ^2^ = 0.188), indicating that the probability of responding that a stimulus was on‐beat increases with BTA, as expected. This means that the odds that a participant responds that the stimulus is on‐beat when BTA = 1 (an on‐beat stimulus) are 22.16 times larger than the odds that a participant responds that the stimulus is on‐beat when BTA = 0 (i.e., an anti‐phase stimulus). To facilitate interpretation, Figure 2A shows the marginal predicted values in terms of probability for Model 2.

The global score on the musical reward scale (BMRQ) ranges from 49 to 96 with *M* = 72.72 and SD = 13.41 for this experiment. Model 3 extended Model 2 by also including BMRQ scores as predictors of the binary response, as well as its interaction with BTA. In addition to the main effect of BTA (*b* = 3.10, *z* = 26.25, *p* < 0.001, *e* ^*b*^ = 22.15), the model indicated a marginally significant interaction between BMRQ and BTA (*b* = 0.20, *z* = 1.743, *p* = 0.081, *e* ^*b*^ = 1.23). No significant main effect of BMRQ emerged (*b* = −0.046, *z* = −0.762, *p* = 0.446, *e* ^*b*^ = 0.95; Marginal *R* ^2^ = 0.085, Conditional *R* ^2^ = 0.188). This interaction suggests a possible tendency for participants with higher BMRQ scores to have a heightened sensitivity to the BTA (i.e., a steeper logistic regression line). Collectively, findings from the behavioral study suggest the effectiveness of the modified task, while simultaneously establishing a robust behavioral model for the TMS investigation.

## 5 Experiment II: Dorsal Versus Medial Right PMC

The objective of Experiment II was to investigate the specific causal involvement of the SMA, pre‐SMA, and dPMC in beat perception. For this purpose, we applied TMS on a defined grid of four locations encompassing the right rostral and caudal dPMC, SMA, and pre‐SMA, and a sham location over the hand area of primary motor cortex (M1). This approach has the advantage to yield a more detailed spatial information on the overall functional organization of the PMC in beat perception (see Lega, Chelazzi, et al. 2020; Lega, Pirruccio, et al. 2020; Cattaneo 2018).

## 6 Materials and Methods

### 6.1 Participants

Forty‐two volunteers took part in the study. Two participants were excluded from the final analysis: the first one because one of the sessions was not recorded due to a technical problem, and the other one because the participant had already participated in Experiment I. After the removal of those subjects, our final sample consisted of 40 participants (16 males, mean age = 23.2 ± 2.37 years). All participants were nonmusicians and had not attended more than three consecutive years of formal music instruction (*M* = 1.00, SD = 1.31). All participants provided written informed consent and completed a TMS safety questionnaire (LRossi et al. 2021). None of the participants reported neurological problems, a history of seizure, and did not present any contraindications related to the use of TMS. The protocol for both TMS experiments was approved by the local ethical committee of the University of Pavia (Ethical Committee Prot. 132/23) and participants were treated in accordance with the Declaration of Helsinki.

### 6.2 Stimuli

Given the TMS experiment's need for a restricted number of trials, we implemented the subsequent adjustments to the task compared to Experiment I. (1) We selected a limited number of BTA, which varied between 0.50 and 0.70 in steps of 0.10 instead of 0.05. We thus considered BTAs = 0.50, 0.60, 0.70 (off‐beat), and 1 (on‐beat). (2) We discarded the five musical tracks from Experiment I with the lowest accuracies with the selected BTAs, thus reducing the number of tracks from 25 to 20. (3) Each musical track was presented once for each BTA, for a total of 80 trials (20 musical tracks × 4 BTAs) per block, corresponding to each of the stimulation sites (see below). This change was implemented in Experiments II and III to have a similar precision in estimating the effects of TMS in all the considered BTA levels.

### 6.3 Neuronavigation

The four active sites of stimulation were located by stereotaxic navigation obtained through a 3D deformation procedure by fitting a high‐resolution MRI model with each individual participant's scalp model derived from 64 points measured on the surface of the head (Softaxic, EMS, Bologna, Italy). This method creates an accurate scalp model without requiring an individual structural MRI, achieving localization accuracy of approximately 5 mm, which is close to that obtained using individual MRI scans (Carducci and Brusco 2012). After reconstructing the 3D brain model, brain surface renderings were used to mark a 4‐point grid covering the dorsal (dPMC) and medial (SMA and pre‐SMA) premotor regions (see Figure 1B). The four points were localized according to individual anatomic landmarks and labeled as points (*P*) from P01 to P04. In particular, P01 (SMA) was located 5 mm lateral to the midline, and 5 mm anterior to the end of the paracentral lobule (Ahdab et al. 2014). P03 (caudal dPMC) was located corresponding to the junction between the precentral sulcus and the superior frontal sulcus (Amiez et al. 2006; Ahdab et al. 2014; Parmigiani et al. 2015; Parmigiani and Cattaneo 2018). P02 (pre‐SMA) and P04 (rostral dPMC) were set by moving 2 cm cranial from P01 and P03 (Ahdab et al. 2014). If any of the initially selected sites elicited a motor‐evoked potential (MEP), we systematically adjusted the placement anteriorly until no MEPs were observed. This ensured that stimulation remained outside the primary motor cortex. As a result, while the targeted premotor sites were highly similar across participants, each individual had a slightly adjusted set of stimulation points. We recorded the exact MNI coordinates of each stimulation site for every participant. Specifically, the mean MNI coordinates for the four points across the sample were: P01: *x* = 5.3, *y* = −3.5, *z* = 77.9; P02: *x* = 4.3, *y* = 16.2, *z* = 71.6; P03: *x* = 29.8, *y* = −5.3, *z* = 71.6; P04: *x* = 27.4, *y* = 16.6, *z* = 65.4. M1 was selected as the sham site and was localized to the primary hand motor area, which was identified by moving the TMS coil around on the scalp, applying single pulses of TMS, and recording muscle responses (see details below). A 3D optical digitizer (Polaris Vicra, NDI) was used in combination with the Softaxic neuronavigation software to co‐register in the same virtual space the participant's head, the digitizer pen, and the TMS coil throughout the whole experiment to monitor coil position for each stimulation location (Lega et al. 2019; Lega, Chelazzi, et al. 2020; Lega, Pirruccio, et al. 2020).

### 6.4 Transcranial Magnetic Stimulation (TMS)

TMS was delivered using a Magstim Rapid2 stimulator (Magstim Co. Ltd. Whitland, UK) connected to a 70‐mm butterfly coil. An online triple‐pulse 10‐Hz (pulse gap of 100 ms) TMS stimulation protocol was employed. This stimulation protocol induces a brief, transient increase in cortical excitability in the target area, as demonstrated by previous studies (Cohen Kadosh et al. 2010; Saad and Silvanto 2013). To determine the appropriate stimulation level for each participant, the resting motor threshold was measured from the first dorsal interosseous muscle of the left hand as determined using the Motor Threshold Assessment Tool, Version 2.0 (www.clinicalresearcher.org/software.htm). An MEP with a peak‐to‐peak amplitude of 50 mV was returned to the software as a valid response. During the experiment, the TMS stimulation was delivered at 100% of the individual motor threshold (average intensity of stimulation *M* = 48.7%, SD = 4.34%). We checked in each participant whether stimulation over the defined 4 premotor sites evoked any MEPs and reassessed the grid if this was the case. For the premotor cortex sites, the coil was oriented at an angle of 45° from the nasion‐inion line and the handle pointing outwards. For the sham condition, the coil was positioned over M1, held perpendicularly in a 90° angle to ensure that the magnetic field did not stimulate the target areas. It has been shown that this sham condition does not produce an electric field capable of modifying neuronal excitability (Lisanby et al. 2001) and has been used in previous studies adopting a similar dense TMS mapping approach (Lega, Chelazzi, et al. 2020; Lega, Pirruccio, et al. 2020).

### 6.5 Electromyography (EMG)

EMG recordings were performed with 10‐mm Ag/AgCl surface electrodes. The active electrode was positioned on the first dorsal interosseous muscle of the left hand and the reference electrode was put on the metacarpophalangeal joint of the index finger. The EMG signal was sampled and amplified 1000 times (1000×) using a Digitimer D360 amplifier (Digitimer) and digitized by an analog‐to‐digital converter (Power 1401, Cambridge Electronic Design) at a sampling rate of 5 kHz, bandpass filtered from 10 to 2 kHz, then stored using Signal (Cambridge Electronic Design) software.

### 6.6 Procedure

Each participant was seated in front of a computer screen (57 cm from the monitor, LCD, 1280 × 1024 Pixel) in a quiet, lighted room and wearing headphones. The procedure for the beat alignment task was identical to Experiment I (see Figure 1A). During the fixation cross, three TMS pulses were delivered immediately before the audio presentation (stimulus onset coincided with the third TMS pulse). Each session consisted of a total of five blocks, corresponding to the five TMS sites (four active sites and one sham condition), with short breaks in between them. The order of the blocks (i.e., the TMS stimulation sites) was counterbalanced across participants. The experiment—stimuli presentation, data collection and TMS triggering—was run by means of the E‐Prime 3.0 Software (Psychology Software Tools, Pittsburgh, PA). At the beginning of the experimental session, participants filled in the BMRQ (Mas‐Herrero et al. 2013). In total, the entire experiment lasted about 2 h.

### 6.7 Statistical Analysis

Based on Experiment I, the data‐analysis strategy relied on multilevel logistic regression models. We first examined potential shifts in participants' responses by predicting the binary response from the trial number (rescaled between 0 and 1 to ensure convergence), considering polynomial effects up to the quartic one. We then used the likelihood‐ratio test and the AIC to compare two alternative models in which the binary response was predicted by BTA and TMS. In the first one (Model 1), we considered only additive effects BTA + TMS. In the second model (Model 2), we also considered the interaction effect between BTA and TMS. Model 1 was designed to test the independent (additive) effects of TMS and BTA on response patterns, while accounting for potential trial progression effects through a third‐degree polynomial of trial number. This allowed us to determine whether each predictor had a significant main effect on participants' responses. Model 2 extended this by including a TMS × BTA interaction term, which was motivated by the hypothesis that the effect of TMS on beat perception might depend on the degree of metronome misalignment (BTA). Given that BTA reflects different levels of beat misalignment, we aimed to assess whether TMS had a uniform effect across all levels of BTA or whether its influence varied depending on how misaligned the metronome was from the actual beat. An additional interaction analysis (Model 3) was carried out to explore whether individual differences in musical reward assessed with the BMRQ were related to the sensitivity of participant responses to BTA and to the TMS. In all models, the effect of TMS was modeled as a series of contrasts between the four TMS stimulation sites and the sham. BTA was centered on its mean and BMRQ was standardized with *M* = 0 and SD = 1, before entering the model. All models included random intercepts per participant and per musical track, to account for both individual differences in response styles and stimulus characteristics. Post hoc tests were carried out using the *phia* package (De Rosario‐Martinez 2015), applying Holm's correction for multiple comparisons (Holm 1979).

## 7 Results

The complete statistics for all models are reported in the Table S4. The first analysis revealed that participants' response patterns significantly changed over the 400 trials, indicating a change in responses across trials. A linear (*b* = −39.750, *z* = −16.877, *p* < 0.001, e^*b*^ = 22.16), quadratic (*b* = 15.176, *z* = 6.545, *p* < 0.001), and cubic (*b* = −7.602, *z* = −3.164, *p* = 0.001) effect of trial number emerged, whereas a quartic effect did not emerge (*b* = 1.153, *z* = 0.510, *p* = 0.610). In light of this finding, we controlled for third‐order polynomial effects of trial number in the subsequent models (Model 1, Model 2 and Model 3).

**FIGURE 3:**
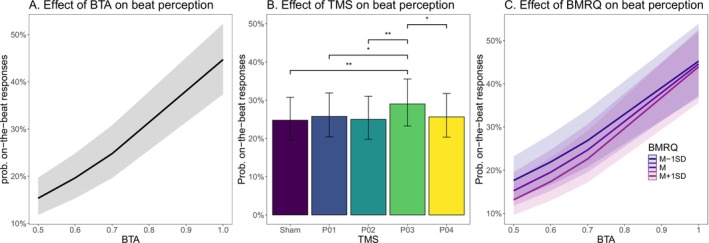
Results of Experiment II. Visualization of the marginal predicted values in terms of on‐beat response probabilities as a function of (A) beep‐track accuracy (BTA) levels, (B) TMS stimulation sites, and (C) of BTA levels and musical reward (BMRQ) score. Error bars in panel B represent the 95% confidence interval.

The likelihood‐ratio test revealed no significant differences in how well Model 1 and Model 2 fitted the data (*ꭓ* ^2^ (4) = 3.93, *p* = 0.415), indicating that considering the interaction effect between BTA and TMS (Model 2) did not improve predictions compared to the model that considers the additive effects of BTA and TMS (Model 1). The same conclusion was suggested by the AIC (AIC_Model1_ = 17,520, AIC_Model2_ = 17,524). Hence, the more parsimonious Model 1 was preferred. A summary of the Model 1 (Marginal *R* ^2^ = 0.106, Conditional *R* ^2^ = 0.230) is illustrated in Table 1. As expected, and in line with Experiment I, the probability of responding that a stimulus was on‐beat increased with BTA. Crucially, stimulating the P03, corresponding to the caudal part of dPMC, significantly increased the probability of indicating that the musical stimulus was on‐beat, compared to the sham control condition. This effect was not observed when TMS stimulation was applied to other sites (see Figure 3B). Post hoc tests confirmed that stimulation of caudal dPMC corresponded to a significantly higher probability of considering a stimulus as on‐beat compared to all other stimulation sites, see Figure 3B (Sham vs. P03: *ꭓ* ^2^ (1) = 13.64, *p* = 0.002; P01 vs. P03: *ꭓ* ^2^ (1) = 7.87, *p* = 0.035; P02 vs. P03: *ꭓ* ^2^ (1) = 12.31, *p* = 0.004; P04 vs. P03: *ꭓ* ^2^ (1) = 8.54, *p* = 0.027). No other significant difference between stimulation sites emerged (all *p*s~1, after Holm correction), see Figure 3B.

**TABLE 1:** Summary of Model 1 of Experiment II.

Predictor	Estimate	Std. error	*Z* value	*p*	*e* ^*b*^
(Intercept)	−3.062	0.168	−18.215	< 0.001	0.05
Trial nr. linear	−42.464	2.358	−18.009	< 0.001	< 0.001
Trial nr. quadratic	16.202	2.385	6.794	< 0.001	1.09*10^7^
Trial nr. cubic	−8.095	2.347	−3.449	< 0.001	< 0.001
TMS P01	0.052	0.059	0.880	0.379	1.05
TMS P02	0.011	0.059	0.194	0.847	1.01
TMS P03	0.216	0.058	3.694	< 0.001	1.24
TMS P04	0.046	0.059	0.777	0.437	1.05
BTA	2.993	0.098	30.521	< 0.001	19.94

Abbreviations: BTA, beep‐track accuracy; TMS P01, SMA; TMS P02, pre‐SMA; TMS P03, caudal dPMC; TMS P04, rostral dPMC (see Figure 1B for a precise localization of the stimulation sites).

The musical reward (BMRQ) scores ranged from 42 to 100 with *M* = 77.83 and SD = 12.23. Model 3 extended Model 2 by including the music reward (BMRQ) score as a predictor. In addition to the main effect of BTA (*b* = 2.996, *z* = 30.553, *p* < 0.001, *e* ^*b*^ = 20.02) and the effect of site 3 TMS stimulation (*b* = 0.219, *z* = 3.753, *p* < 0.001, *e* ^*b*^ = 1.25), the model indicated a significant interaction between BMRQ and BTA (*b* = 0.295, *z* = 2.982, *p* = 0.002, *e* ^*b*^ = 1.34). This interaction indicates that participants with a higher musical reward score are more sensitive to the metronome misalignment (see Figure 3C), increasing our confidence in the trend identified in Experiment I. No significant main effect of BMRQ emerged (*b* = −0.114, *z* = −1.379, *p* = 0.168, *e* ^*b*^ = 0.89) nor an interaction between BMRQ and any of the TMS sites (all *ps* > 0.453, all *e* ^*b*^ < 1.05; Marginal *R* ^2^ = 0.109, Conditional *R* ^2^ = 0.231).

## 8 Experiment III: Preregistered Test of Lateralization and Replication of Experiment II

Results of the Experiment II indicated that the selective stimulation of the caudal part of the dPMC in the right hemisphere significantly affected participants' beat perception. Therefore, the main aim of Experiment III was to test whether the role of caudal dPMC in beat perception was specific to the right hemisphere. In addition, this experiment served as a replication of the original result in an independent sample. To do this, we used the same task and TMS procedure employed in Experiment II, but with stimulation only over right and left dPMC and the sham location. Experiment III was preregistered on the AsPredicted platform (https://aspredicted.org/D7B_9CK).

## 9 Materials and Methods

### 9.1 Participants

To estimate power required to detect the crucial main effect of right dPMC TMS (vs. Sham) for Experiment III, we used the simulation approach implemented in the R package *simr* (Green and MacLeod 2016). The simulation was based on the results of Experiment II. All parameters were set to the values that emerged in Experiment II, with the exception of the crucial effect, for which we applied a *safeguard power* approach (Perugini et al. 2014): instead of powering the study to detect the original effect (*b* = 0.216, see Table 1), we powered it to detect a smaller effect, corresponding to the lower bound of the 60% confidence interval (*b* = 0.167). The results suggested that *N* = 42 participants would provide 82.5% power to detect the crucial effect.

Thus, 42 volunteers took part in the study (17 males, mean age = 23.5 ± 2.61 years). All participants were nonmusicians and had not received more than three consecutive years of formal music instruction (*M* = 0.90, SD = 1.28). Prior to the TMS experiment, participants provided written informed consent and completed a questionnaire to assess their suitability for TMS administration (adapted from Rossi et al. 2021). None of the participants had a history of neurological issues or seizures, and there were no contraindications for TMS.

### 9.2 Procedure

Stimuli, TMS (average intensity of stimulation *M* = 48.1%, SD = 7.6%), EMG, and procedure were identical to Experiment II (see Figure 1A). In the sham condition, the coil was positioned over the vertex, held perpendicularly at a 90° angle. Mean MNI coordinates for Experiment III were *x* = 28.9, *y* = −5.8, *z* = 72.9 for right dPMC and *x* = −31.8, *y* = −3.8, *z* = 71.4 for left dPMC (see Figure 1C). To directly compare the right dPMC stimulation sites between Experiment II and III, we quantify their spatial similarity by calculating the Euclidean distance between the mean stimulation locations. The resulting Euclidean distance of 1.62 mm indicates a high degree of overlap between the stimulation sites across the two experiments (see also Supporting Information, Table S1 and Figure S1).

### 9.3 Statistical Analysis

The following statistical analysis was preregistered to examine the main hypothesis: we fitted a multilevel logistic regression model (Model 1), in which the response (0 = off‐beat, 1 = on‐beat) was predicted by BTA and TMS. Based on the results of Experiment II, we took into account learning effects by including linear, quadratic, and cubic effects of time, and we considered BTA as a continuous predictor ranging between 0.50 and 1. We included random intercepts per participant and per musical track. Our main hypothesis was the replication of a positive main effect of right dPMC TMS stimulation vs. Sham (one‐tailed)^1^. We also examined the effects of lateralization. We performed an additional interaction analysis (Model 2) to investigate whether individual differences in musical reward assessed with the BMRQ were related to the sensitivity of participants' responses to BTA.

## 10 Results

**FIGURE 4:**
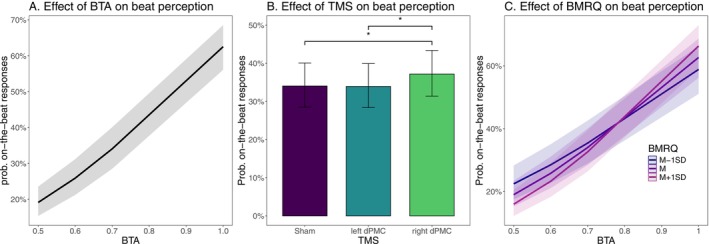
Results of Experiment III. Visualization of the marginal predicted values in terms of on‐beat response probabilities as a function of (A) beep‐track accuracy (BTA) levels, (B) TMS stimulation sites, and (C) BTA levels and music reward (BMRQ) score. Error bars in panel B represent the 95% confidence interval.

The complete statistics for all models are reported in the Table S5. A summary of Model 1 (Marginal *R* ^2^ = 0.149, Conditional *R* ^2^ = 0.239) is illustrated in Table 2. In line with Experiment I and II, Model 1 revealed a significant effect of BTA: as expected, the probability of responding that a stimulus was on‐beat increased with BTA. Crucially, the model confirmed our main hypothesis by revealing that stimulating the right dPMC significantly increased the probability of indicating that the musical stimulus is on‐beat compared to the sham control condition (*b* = 0.136, *z* = 2.443, *p* = 0.007, *e* ^*b*^ = 1.15). Model 1 also showed that the effect of stimulating the left dPMC did not significantly differ from the sham control condition, see Figure 4B and Table 2. Post hoc tests (Holm correction) confirmed that stimulation of the right dPMC corresponded to a significantly higher probability of considering a stimulus as on‐beat compared to both Sham (*ꭓ* ^2^ (1) = 5.96, *p* = 0.032) and left dPMC (*ꭓ* ^2^ (1) = 6.47, *p* = 0.032). No significant difference was found between Sham and left dPMC stimulation (ꭓ^2^ (1) = 0.009, *p* = 0.92), see Figure 4B.

**TABLE 2:** Summary of Model 1 of Experiment III.

Predictor	Estimate	Std. error	*Z* value	*p*	*e* ^*b*^
(Intercept)	−3.402	0.157	−21.619	< 0.001	0.03
Trial nr linear	−31.332	2.376	−13.184	< 0.001	< 0.001
Trial nr quadratic	−0.627	2.333	−0.269	0.786	0.53
Trial nr cubic	−7.789	2.348	−3.317	< 0.001	< 0.001
TMS Left	−0.006	0.056	−0.099	0.921	0.99
TMS Right	0.136	0.056	2.443	0.007^a^	1.15
BTA	3.907	0.124	31.582	< 0.001	49.76

^a^ Because we preregistered a directional hypothesis (a positive effect of the right TMS stimulation), the *p*‐value reported is one‐tailed. The two‐tailed *p*‐value is *p* = 0.015.

The musical reward (BMRQ) scores in this experiment ranged from 57 to 97 with *M* = 80.43 and SD = 8.75. Model 2 extended Model 1 by including as predictors of the binary response also the overall BMRQ score as well as its interaction with BTA and with TMS. Results confirmed the main effect of BTA (*b* = 3.938, *z* = 31.654, *p* < 0.001, *e* ^b^ = 51.33), and the effect of right dPMC stimulation (*b* = 0.139, *z* = 2.492, *p* = 0.013, *e* ^b^ = 1.15). Furthermore, the model indicated a significant interaction between BMRQ and BTA (*b* = 0.740, *z* = 6.135, *p* < 0.001, *e* ^b^ = 2.09). There was no statistically significant main effect of BMRQ (*b* = −0.062, *z* = −0.826, *p* = 0.410, *e* ^b^ = 0.39), and similarly, no significant interaction was observed between BMRQ and right dPMC TMS (*b* = 0.021, *z* = 0.378, *p* = 0.710, *e* ^b^ = 1.02) or left dPMC TMS (*b* = 0.056, *z* = 1.004, *p* = 0.320, *e* ^b^ = 1.06; Marginal *R* ^2^ = 0.154, Conditional *R* ^2^ = 0.245). In line with Experiments I and II, the interaction between BMRQ and BTA indicated that participants with a higher musical reward exhibited better performance and higher rhythm perceptual abilities (see Figure 4C).

## 11 Discussion

This study adopted a systematic, multifocal topographical causal approach to comparatively assess the contribution of premotor regions in the auditory dorsal stream in musical beat perception. Results indicated that inhibitory stimulation of the caudal portion of right dPMC selectively affected beat perception compared to stimulation of pre‐SMA, SMA, or the left dPMC. rTMS over right dPMC impaired the ability to detect small temporal deviations. These results are in line with the predictions of recent models that emphasize the role of the dorsal auditory stream in auditory beat‐based timing perception (Patel and Iversen 2014; Proksch et al. 2020; Ross et al. 2018b; Morillon and Baillet 2017; Cannon and Patel 2021). They also provide further support for the dorsal premotor circuits as a crucial node for internal action predictions and for the generation of perceptual expectations of ongoing sequential events (Schubotz and von Cramon 2002). Furthermore, the current results align with the dominant role of the right dorsal stream in music perception (Zatorre and Belin 2001; Johnsrude et al. 2000; Patterson et al. 2002; Warrier and Zatorre 2004; Janata 2015) and auditory‐motor integration in the context of music (Vaquero et al. 2018; Moore et al. 2017; Lega et al. 2016; Siman‐Tov et al. 2022; Jünemann et al. 2023), including musical rhythm perception (Chen et al. 2008a) and production (Giovannelli et al. 2014). Finally, across all three experiments, we consistently demonstrated that individual differences in musical reward sensitivity, as assessed by the BMRQ, predicted sensitivity to the beat misalignment, providing additional evidence supporting the association between music reward and rhythmic abilities (Fiveash et al. 2022, 2023).

Beat perception in humans is inherently predictive (Penhune and Zatorre 2019; Cannon and Patel 2021; Patel and Iversen 2014; Grahn and Rowe 2013). Neuroimaging studies have consistently shown the engagement of motor planning regions of the dorsal stream in beat perception, even in the absence of movement (Grahn and Brett 2007; Chen et al. 2008a, 2008b; Gordon et al. 2018). Our current findings align nicely with the idea that motor planning regions in the human brain, including PMC, are critical in generating top‐down temporal predictions to guide perception (Arnal 2012; Patel and Iversen 2014; Hadley et al. 2015; Ross et al. 2016; Morillon and Baillet 2017; Araneda et al. 2017). In this context, one possibility is that in the current study, dPMC stimulation enhanced reliance on top‐down expectations, making participants less sensitive to subtle timing mismatches and more likely to categorize slightly misaligned beats as on time. Alternatively, stimulation may have altered prediction error processing, reducing sensitivity to small deviations and biasing participants toward perceiving stimuli as more on‐beat, even when they were misaligned. Notably, our study establishes, for the first time, a direct link between beat perception and a specific portion of the dorsal PMC. In a multimodal imaging study examining the anatomical and functional parcellation of the PMC, Genon et al. (2017) identified five subregions that differed in their connectivity and relationships with cognitive tasks. The coordinates of the caudal dPMC region in our study lie at the junction of Genon's central and dorsal clusters. These clusters are associated with motor and cognitive functions supporting sequencing and rhythm processing, which are relevant for finger movements, music, and language (Genon et al. 2017, refer to Figure S1). Similarly, two TMS studies targeting the caudal superior frontal gyrus (Cattaneo and Parmigiani 2021; Tagliaferri et al. 2023) showed that this region is crucial for internal timing of actions in predictive behavior. Therefore, the human dPMC seems to exhibit functional characteristics, including temporal prediction and the generation of temporal patterns, akin to those expected in the SMA of non‐human primates. Given contemporary models of beat perception that highlight the role of SMA are derived from non‐human primate studies (Merchant and Honing 2014; Merchant et al. 2015; Merchant and Averbeck 2017; Cannon and Patel 2021), it remains a possibility that this function in humans is localized more laterally in the PMC.

Our results show that inhibitory stimulation over pre‐SMA/SMA does not impact beat perception. This result is at odds with recent preliminary findings reporting that tDCS over SMA, but not dPMC, influenced rhythm discrimination (Leow et al. 2022), and with a large number of neuroimaging studies that consistently show activation of the SMA during beat perception and synchronization (Chen et al. 2008a, 2008b; Grahn and Brett 2007; Grahn and Rowe 2009, 2013; Grahn and Schuit 2012; Kung et al. 2013; Matthews et al. 2020). There are several reasons why this might be the case. First, our dPMC coordinates are more medial compared to those used by Leow et al. (2022). Given the lower spatial resolution of tDCS compared to TMS, it is possible that the SMA stimulation in this study also affected the caudal dPMC region identified in our study. Further, their dPMC stimulation was more ventral and not directly targeted by the grid used in our current experiment. Second, although we maintained consistent stimulation intensity across the five points to reduce variability in the stimulation parameters, it is plausible that we were unable to effectively reach the SMA with TMS due to its deeper location compared to the other premotor points. Third, it may be that the dPMC and SMA play different roles in different aspects of beat‐based timing perception (Ross et al. 2018b). The process of beat perception has been hypothesized to comprise two subprocesses (Cameron and Grahn 2016; Grahn and Rowe 2013; Toiviainen et al. 2020): first, beat finding, which refers to the active inference of perceived beat; and second, beat maintenance or beat continuation, which refers to the ongoing series of predictions based on the internal model of the beat derived during beat finding. There is some evidence supporting a neural dissociation of these two processes with the basal ganglia, in particular the putamen and its connection to SMA, responding significantly more to conditions associated with beat continuation than beat finding (Grahn and Rowe 2013). Further, studies in non‐human primates that demonstrate the role of the SMA in beat processing all use paradigms where the animal must maintain and continue the beat of a given stimulus (Merchant et al. 2015). The beat perception task used in the present experiments likely relies more on beat finding, mediated by the dPMC, as the participant must extract the beat of each excerpt to decide if the overlaid beat track is on or off. Conversely, the basal ganglia‐SMA connection may be involved in more implicit or over‐learned predictions of regularity rather than task‐based attention to prediction (Grahn and Rowe 2013; Kung et al. 2013). Basal ganglia activity is observed even when participants are not directed to attend to the beat (Grahn and Rowe 2009), whereas previous evidence indicated that PMC activation is present when attention is directed towards prediction, but not when temporal regularity is present yet unattended (Schubotz et al. 2000). Therefore, in explicit beat‐finding tasks like the one used in the current study, participants may rely more on motor representations supported by the dPMC. This hypothesis warrants further investigation in future studies.

Our results also demonstrate that the engagement of the dPMC in beat‐based timing perception is lateralized to the right hemisphere. Evidence on laterality in rhythm perception and perceptual timing tasks is mixed (see Kasdan et al. 2022 for a review). Previous neuroimaging (Chen et al. 2008a) and brain stimulation studies (Ross et al. 2018a, 2018b) found bilateral or greater left hemisphere involvement in beat perception. Ross et al. (2018a) found that cTBS over the left PPC impaired participants' ability to make phase judgments about metronome alignment with musical beats, while their stimulation of the left dPMC disrupted tempo (but not phase) perception. Notably, they did not investigate the role of the right dPMC. In contrast, our study showed that repetitive TMS (rTMS) over the right, but not left, dPMC affected phase perception. These seemingly disparate results likely reflect the distinct contributions of different regions within this network, as well as methodological variations between studies. While Ross et al. (2018a, 2018b) employed an adaptive version of the beat alignment test (BAT), adjusting stimulus difficulty based on individual performance, our paradigm relied on a fixed set of stimuli consistent across participants. Furthermore, their use of cTBS, which induces longer‐lasting inhibitory effects, contrasts with our application of online rTMS, which transiently disrupts ongoing neural processing. Although we did not test tempo perception, the selective effect on phase processing following right dPMC stimulation indicates that this region may be particularly involved in the real‐time tracking and integration of temporal cues with motor representations. In contrast, left‐lateralized regions, as implicated in Ross et al. (2018a, 2018b) may contribute more to explicit, cognitively demanding comparisons, such as those involved in timing discrimination interval or in adaptive judgment tasks. This functional specialization may be underpinned by neuroanatomical differences, as highlighted by Schneider et al. (2023). Their work suggests that the right planum temporale (PT), acting as an “integrational hub” for auditory information due to its greater myelination and bilateral integration capacity, could play a crucial role in providing robust auditory input to right‐hemisphere motor regions, including the dPMC. At the same time our findings fit with other studies showing a predominant role of the right dPMC, as opposed to the left, both in musical rhythm perception (Chen et al. 2008a) and production (Giovannelli et al. 2014; Kung et al. 2013). Notably, the arcuate fasciculus which connects brain regions of the dorsal auditory stream, has been associated with musical auditory‐motor feedback, particularly in the right hemisphere (Vaquero et al. 2018; Moore et al. 2017; Jünemann et al. 2023). This outcome can be understood in terms of the dominant role of the right dorsal stream in auditory‐motor integration, particularly within the context of music (see Jünemann et al. 2023). Additionally, Vaquero et al. (2018) demonstrated that the volume of the right, but not the left, AF predicted synchronization improvement during a rhythmic task. Another interpretation of this right‐hemispheric dominance is associated with its specific involvement in implicit versus explicit perceptual timing processes. Coull and Nobre (2008) suggested higher activity on the right side for explicit timing and greater left‐sided activity for more implicit timing. In this context, we hypothesize that participants in the current experiment engaged in a more explicit and direct comparison of timing intervals in the musical stimuli to perform the task, thus exhibiting a stronger TMS effect when the stimulation was applied to the right dPMC. Finally, dPMC lateralization appears to be particularly sensitive to individual differences and expertise. For example, prior studies have indicated a more robust activation of the right dPMC in nonmusicians compared to expert musicians during synchronization tasks (Chen et al. 2008a; Limb et al. 2006). Additionally, Grahn and McAuley (2009) observed stronger activation of the right PMC in weak‐beat perceivers and of the left PMC in strong‐beat perceivers. Investigating the causal involvement of the left versus right dPMC in rhythm perception, considering interindividual factors, could be an additional avenue for future studies.

A limitation of our study is that our data, taken in isolation, may not rule out the possibility that dPMC stimulation results in a non‐specific perceptual bias. However, a previous study that applied the same stimulation approach to the same area during a very different task did not reveal any such effect (Lega, Chelazzi, et al. 2020). Rather, a body of studies strongly supports the involvement of the dPMC in rhythm perception and performance (Chen et al. 2008a; Grahn and Brett 2007; Bengtsson et al. 2009; Grahn and Rowe 2013). Therefore, while future studies will be valuable in further clarifying this issue, the available evidence suggests that the observed effect is more likely to be specific to rhythm processing rather than reflecting a non‐specific perceptual bias.

Finally, in three different experiments we consistently demonstrated a relation between individual differences in musical reward sensitivity and rhythmic abilities, so that higher scores on the BMRQ were related to better sensitivity in rhythm perception. We hypothesize that, despite our sample consisting of nonmusicians, participants with higher music reward scores may engage with music more frequently and participate in musical activities more often. This increased exposure could contribute to a heightened sensitivity to rhythmic structures, refining their perceptual abilities and making them more attuned to subtle variations in timing. To the best of our knowledge, only one paper has directly investigated the effect of individual differences in musical reward tested with the BMRQ and rhythmic abilities (Fiveash et al. 2022), but they failed to find a significant effect. Importantly, in this study the authors employed a median split approach to investigate whether the BMRQ could predict whether participants were classified as weak or strong performers (Fiveash et al. 2022). In contrast, we adopted a more sensitive trial‐level approach that may have allowed us to detect this relationship. These results pave the way for future investigations into the influence of musical reward on both production and perceptual rhythmic abilities. Moreover, considering the co‐activation of the motor and reward networks during the sensation of groove (Matthews et al. 2020; Zalta et al. 2024; Romkey et al. 2025; Spiech et al. 2022, 2024), it would be interesting to specifically examine the role of dPMC in beat‐based timing perception in relation to different degrees of rhythm complexity. Additionally, while our study focused on nonmusicians, future research should explore the interplay between musical reward sensitivity, musical training, and rhythm perception. The link between rhythmic perceptual abilities and musical training has been well established using both the original and adaptive versions of the BAT (Iversen and Patel 2008; Harrison and Müllensiefen 2018a; Spiech et al. 2023), as well as other rhythmic perception tasks (Matthews et al. 2016). Notably, this relationship persists regardless of regular practice or active engagement with music (Spiech et al. 2023) and remains independent of the type of first instrument played (Matthews et al. 2016). Moreover, prior research has demonstrated a strong association between musical training and musical reward sensitivity, with higher levels of training corresponding to greater BMRQ scores (Mas‐Herrero et al. 2013). However, the relationship between these three factors—musical reward sensitivity, musical training, and rhythm perception—has yet to be fully examined. Investigating this interaction could provide deeper insights into the mechanisms underlying individual differences in rhythm perception and musical engagement.

In conclusion, in two different TMS stimulation studies we demonstrated that the right caudal portion of the dPMC is directly involved in beat‐based timing perception. This effect was topographically specific because no effect on a BAT was observed with TMS of the left dPMC, of the SMA and pre‐SMA, or of a control site. These findings are consistent with models proposing that motor planning regions in the dorsal auditory stream play an active role in rhythm perception (Cannon and Patel 2021; Ross et al. 2016, 2018a, 2018b; Patel and Iversen 2014; Morillon and Baillet 2017). Accordingly, the perception of musical beat is seemingly a powerful example of the neuronal recycling hypothesis originally proposed by Dehaene and Cohen (2007) in which culturally dependent brain functions take advantage of neural circuits that evolved for other purposes. In this case, the relatively novel brain capacity for musical beat perception would rely on a phylogenetically ancient brain circuit, that is, the one that is used to produce accurately timed movements. While this suggests that the neural circuitry underlying musical beat perception may be rooted in ancestral motor systems, it is important to acknowledge that spontaneous predictive synchronization to a metronome, as observed in humans, may not be a ubiquitous feature across primates. Studies have shown that monkeys, while capable of synchronizing their movements to a metronome, may primarily exhibit reactive rather than predictive synchronization (Takeya et al. 2017). This finding suggests that the human capacity for predictive beat perception may involve unique cognitive and neural mechanisms that have evolved alongside complex social and cultural interactions, such as those involved in music making and dance (Lenc et al. 2021; Brown 2022; Honing 2019; Patel and Iversen 2014). Together our findings significantly contribute to contemporary models of timing perception, underscoring the distinct roles that various cortical areas within motor control networks play in the prediction and perception of musical rhythm.

## Author Contributions

**C.L**., **L.C**., and **V.P**. conceived the study and the behavioral paradigms. **C.L**., **L.C**., and **V.P**. developed the paradigm. **G.L**., **A.M**., and **S.L.R**. collected the data. **C.L**. and **G.C**. analyzed the data. All the authors discussed the findings and provided valuable insights as to their interpretation. **G.L**. and **C.L**. wrote the paper.

## Conflicts of Interest

The authors declare no conflicts of interest.

## Supporting information


**Data S1.**hbm70225‐sup‐0001‐supinfo.

## Data Availability

An anonymized copy of the preregistration of Experiment III is available at https://aspredicted.org/D7B_9CK. Data and R code for reproducing our results are available at https://osf.io/atkxp/?view_only=66a3e248d286491e88d89f2dd3072e9a.

## Endnotes

^1^
For the sake of clarity, in the pre‐registration, we coded the dependent variable “Response” in the opposite direction, where 1 represented “off‐beat” and 0 represented “on‐beat.” Consequently, the pre‐registration indicated a negative effect of dPMC stimulation. By coding the Response variable as 0 for “off‐beat” and 1 for “on‐beat” here, we anticipated a positive effect of TMS stimulation. This adjustment represents a minor deviation from the initial pre‐registration.
