# Supplementary material for: Topography of Functional Organization of Beat Perception in Human Premotor Cortex: Causal Evidence From a Transcranial Magnetic Stimulation (TMS) Study
Source: Hum Brain Mapp. 2025 May 9;46(7):e70225. doi: 10.1002/hbm.70225 (PMC12063481; doi:10.1002/hbm.70225)
Supplement: Supplementary file 1 — Data S1.hbm70225‐sup‐0001‐supinfo. [file HBM-46-e70225-s001.docx]

**Supplementary Materials**

*Table S1.* Accuracy data for each BTA level across the three experiments.

|  | EXP.I | | EXP.II | | EXP.III | |
| --- | --- | --- | --- | --- | --- | --- |
|  | *Mean* | *SD* | *Mean* | *SD* | *Mean* | *SD* |
| BTA = 0.5 | .599 | .491 | .778 | .415 | .758 | .428 |
| BTA = 0.55 | .566 | .496 | / | / | / | / |
| BTA = 0.6 | .531 | .499 | .745 | .436 | .733 | .443 |
| BTA = 0.65 | .491 | .500 | / | / | / | / |
| BTA = 0.7 | .455 | .498 | .705 | .456 | .651 | .477 |
| BTA = 0.75 | .430 | .495 | / | / | / | / |
| BTA = 0.8 | .415 | .493 | / | / | / | / |
| BTA = 1 | .734 | .442 | .505 | .500 | .630 | .483 |

*Table S2.* Mean MNI coordinates of the caudal dPMC stimulation sites in Experiment II and III. To quantify their similarity, we performed independent t-tests comparing the x, y, and z coordinates between the two experiments. None of the comparisons were statistically significant (all ps > .22) indicating no meaningful differences in stimulation sites, see also Figure S2.

| Experiment | x | | y | | z | |
| --- | --- | --- | --- | --- | --- | --- |
|  | *Mean* | *SD* | *Mean* | *SD* | *Mean* | *SD* |
| II | 29.82 | 1.78 | -5.32 | 2.54 | 71.62 | 2.36 |
| III | 28.92 | 1.70 | -5.76 | 2.89 | 72.90 | 1.30 |

*Figure S1.* Graphical representation of Table S1.


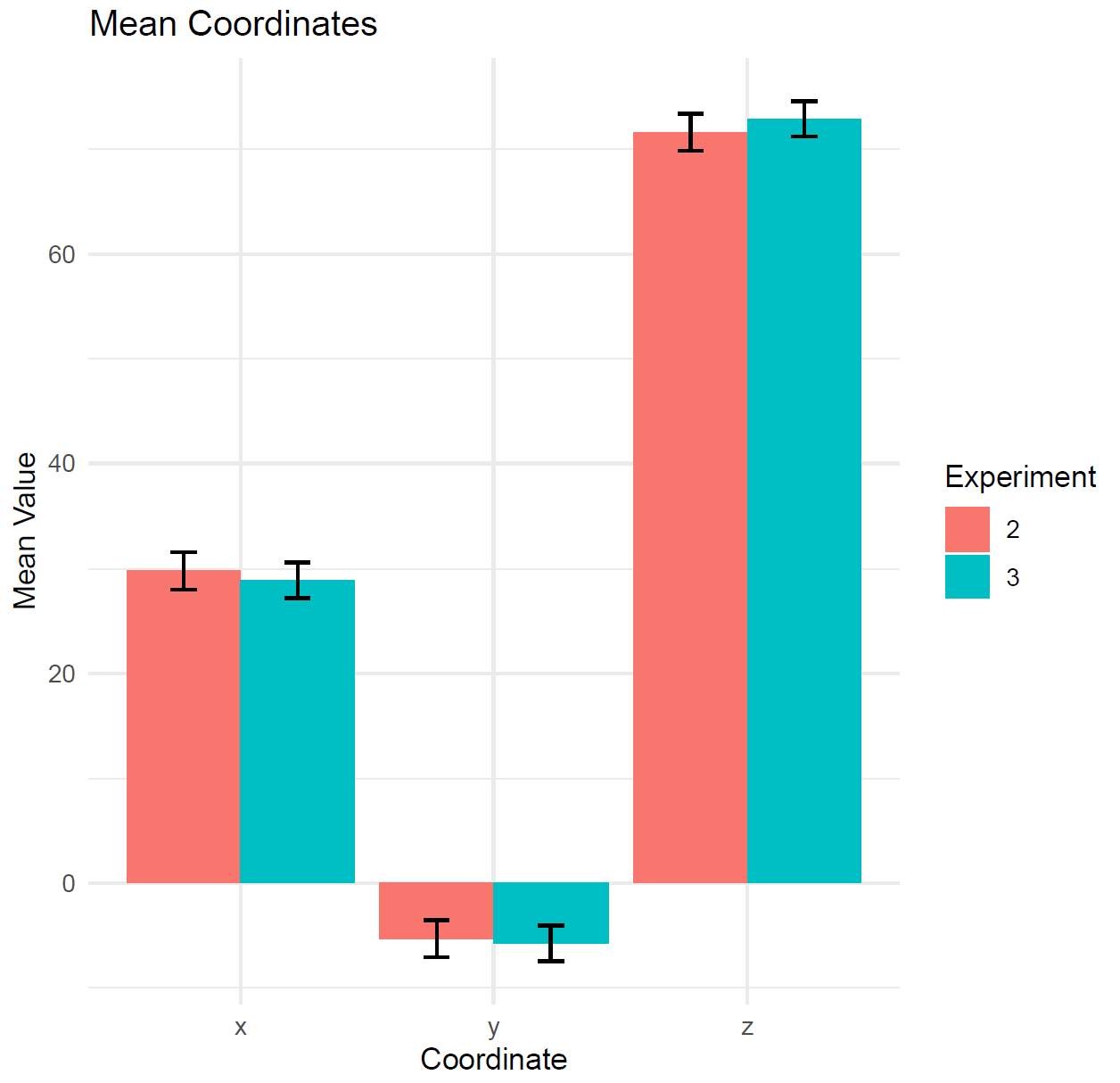


**Experiment I**

*Table S3.* Overall fit of all the models in Experiment I. BTA = beep-track accuracy, BMRQ = Barcelona Music Reward Questionnaire.

| Model 2 | | | |
| --- | --- | --- | --- |
|  | ꭓ2 | df | p-value |
| BTA | 689.51 | 1 | <.001 |
| Model 3 | | | |
|  | ꭓ2 | df | p-value |
| BTA | 688.59 | 1 | <.001 |
| BMRQ | 0.66 | 1 | .414 |
| BMRQ x BTA | 3.04 | 1 | .081 |

**Experiment II**

*Table S4.* Overall fit of all the models in Experiment II. BTA = beep-track accuracy, BMRQ = Barcelona Music Reward Questionnaire.

| Model 1 | | | |
| --- | --- | --- | --- |
|  | ꭓ2 | df | p-value |
| Learning | 393.52 | 3 | <.001 |
| BTA | 931.35 | 1 | <.001 |
| TMS | 18.08 | 4 | .001 |
| Model 3 | | | |
|  | ꭓ2 | df | p-value |
| Learning | 372.27 | 3 | <.001 |
| BTA | 929.95 | 1 | <.001 |
| TMS | 18.29 | 4 | .001 |
| BMRQ | 1.46 | 1 | .226 |
| BMRQ x BTA | 8.89 | 1 | .003 |
| BMRQ x TMS | 1.41 | 4 | .843 |

**Experiment III**

*Table S5.* Overall fit of all the models in Experiment III. BTA = beep-track accuracy, BMRQ = Barcelona Music Reward Questionnaire.

| Model 1 | | | |
| --- | --- | --- | --- |
|  | ꭓ2 | df | p-value |
| Learning | 197.62 | 3 | <.001 |
| BTA | 998.37 | 1 | <.001 |
| TMS | 8.33 | 2 | .016 |
| Model 2 | | | |
|  | ꭓ2 | df | p-value |
| Learning | 182.33 | 3 | <.001 |
| BTA | 978.91 | 1 | <.001 |
| TMS | 8.43 | 2 | .015 |
| BMRQ | 0.18 | 1 | .673 |
| BMRQ x BTA | 37.64 | 1 | <.001 |
| BMRQ x TMS | 1.03 | 2 | .597 |
